# Emergence of plasmid-mediated colistin resistance *mcr*-3.5 gene in *Citrobacter amalonaticus* and *Citrobacter sedlakii* isolated from healthy individual in Thailand

**DOI:** 10.3389/fcimb.2022.1067572

**Published:** 2023-01-04

**Authors:** Thanawat Phuadraksa, Sineewanlaya Wichit, Napat Songtawee, Srisurang Tantimavanich, Chartchalerm Isarankura-Na-Ayudhya, Sakda Yainoy

**Affiliations:** ^1^ Department of Clinical Microbiology and Applied Technology, Faculty of Medical Technology, Mahidol University, Nakhon Pathom, Thailand; ^2^ Department of Clinical Chemistry, Faculty of Medical Technology, Mahidol University, Nakhon Pathom, Thailand

**Keywords:** colistin resistance, citrobacter spp., citrobacter sedlakii, citrobacter amalonaticus, mcr gene, mcr-3

## Abstract

*Citrobacter* spp. are Gram-negative bacteria commonly found in environments and intestinal tracts of humans and animals. They are generally susceptible to third-generation cephalosporins, carbapenems and colistin. However, several antibiotic resistant genes have been increasingly reported in *Citrobacter* spp., which leads to the postulation that *Citrobacter* spp. could potentially be a reservoir for spreading of antimicrobial resistant genes. In this study, we characterized two colistin-resistant *Citrobacter* spp. isolated from the feces of a healthy individual in Thailand. Based on MALDI-TOF and ribosomal multilocus sequence typing, both strains were identified as *Citrobacter sedlakii* and *Citrobacter amalonaticus*. Genomic analysis and S1-nuclease pulsed field gel electrophoresis/DNA hybridization revealed that *Citrobacter sedlakii* and *Citrobacter amalonaticus* harbored *mcr-3.5* gene on pSY_CS01 and pSY_CA01 plasmids, respectively. Both plasmids belonged to IncFII(pCoo) replicon type, contained the same genetic context (Tn*3-*IS*1-*ΔTnAs*2-mcr-3.5-dgkA-*IS*91*) and exhibited high transferring frequencies ranging from 1.03×10^-4^ - 4.6×10^-4^ CFU/recipient cell *Escherichia coli* J53. Colistin-MICs of transconjugants increased ≥ 16-fold suggesting that *mcr-3.5* on these plasmids can be expressed in other species. However, beside *mcr*, other major antimicrobial resistant determinants in multidrug resistant Enterobacterales were not found in these two isolates. These findings indicate that *mcr* gene continued to evolve in the absence of antibiotics selective pressure. Our results also support the hypothesis that *Citrobacter* could be a reservoir for spreading of antimicrobial resistant genes. To the best of our knowledge, this is the first report that discovered human-derived *Citrobacter* spp. that harbored *mcr* but no other major antimicrobial resistant determinants. Also, this is the first report that described the presence of *mcr* gene in *C. sedlakii* and *mcr-3* in *C. amalonaticus*.

## Introduction

The emergence of antimicrobial resistance (AMR) is one of the most public health concerns. As declared by the World Health Organization (WHO) recently, the most problematic multidrug-resistant (MDR) bacteria is carbapenem-resistant Gram-negative bacilli, in particular Enterobacterales (CRE) (Tacconelli et al., 2018). The presence of such MDR bacteria and the lack of new antimicrobial agents lead to the use of colistin, which has been considered as a last-resort antibiotic (Madec et al., 2017; Zheng et al., 2020; Ouchar Mahamat et al., 2021). Colistin is a cyclic polypeptide antibiotic that targets the lipid A moiety of lipopolysaccharide (LPS), causing destabilization of the bacterial outer membrane, and leading to cell death. Beside clinical usage, colistin was also heavily used as a growth promoter in livestock (Rahal, 2008). As a result of the increased use in clinical practice and inappropriate use in animal production, acquired colistin resistance has emerged (Rahal, 2008; Papp-Wallace et al., 2011). Most of colistin resistant mechanisms are related to chromosomal mutation within two-component systems (TCSs), resulting in modification of LPS by addition of positively charged molecules including phosphoethanolamine (PEtN) and 4-amino-4-deoxy-L-arabinose (Ara4N) to the 1-phosphate or 4-phosphate groups of Lipid A, respectively. Beside chromosomal mutations, plasmid-mediated mobile colistin resistant (*mcr*) gene has also been reported. The discovery of *mcr-1* in 2015 has raised a significant public health concern, since the gene can easily spread by horizontal gene transfer (Liu et al., 2016). Shortly after the discovery of *mcr*-1, other genetic alleles including *mcr-2* to *mcr-10* have been identified from various species of Gram-negative bacteria (Xavier et al., 2016; Yin et al., 2017; Borowiak et al., 2017; AbuOun et al., 2018; Wang et al., 2018; Yang et al., 2018; Kieffer et al., 2019; Wang et al., 2020). Currently, *mcr* genes have been distributed globally. The genes have been identified in at least 70 countries, with *mcr*-1 being the most prevalent followed by *mcr*-3 and *mcr*-4, respectively. They are frequently isolated from *E. coli*, *K. pneumoniae* and *Salmonella* spp. (Mmatli et al., 2022). In Thailand, various *mcr* alleles including *mcr*-1, *mcr*-2, *mcr*-3, *mcr*-6, *mcr*-7, *mcr*-8, and *mcr*-9 have been reported. Most of these alleles were found to associate with farmed animals, especially pig and poultry (Mmatli et al., 2022). Beside animals, prevalence of *mcr*-1 in human patients and co-occurrence of *mcr* -2 and *mcr* -3 on chromosome of multidrug-resistant *Escherichia coli* isolated from a healthy subject were recently reported by our group (Eiamphungporn et al., 2018; Phuadraksa et al., 2022).

*Citrobacter* spp. are Gram-negative bacteria in the order Enterobacterales. It is commonly found in soil, water, retail meat, and intestines of animals and humans (Liu et al., 2018b). It has been reported to carry several types of antimicrobial resistant genes such as AmpC *β*-lactamase, extended-spectrum -lactamases, plasmid-mediated quinolone resistant determinants, and carbapenemases (Jacobson et al., 1995; Hanson and Sanders, 1999; Wang et al., 2000; Mohanty et al., 2007; Zhang et al., 2008; Samonis et al., 2009; Shahid, 2010; Kanamori et al., 2011; Lee et al., 2015). Moreover, several variants of *mcr* genes have been recently reported in many species of *Citrobacter*, including *mcr-1* (Li et al., 2017; Hu et al., 2017; Zhou et al., 2017; Sadek et al., 2021) and *mcr-9* (Bitar et al., 2020) in *C*. *freundii*, *mcr-1* in *C*. *braakii* (Sennati et al., 2017; Liu et al., 2018a; Zelendova et al., 2020), and *mcr-1.5* in *C*. *amalonaticus* (Faccone et al., 2019). Therefore, *Citrobacter* spp. has been speculated as a potential source for carrying and spreading of antibiotic resistant genes (Jiang et al., 2019).

Herein, colistin-resistant *C. sedlakii* and *C. amalonaticus* were isolated from healthy individual under healthcare check-ups program at the Golden Jubilee Medical Center Mahidol University, Nakhon Pathom, Thailand, in 2022. The antimicrobial susceptibility profile, whole genome sequencing, AMR mechanisms, plasmid characteristics and transferring frequencies were investigated.

## Materials and methods

### Bacterial identification and isolation of colistin-resistant *Citrobacter* strains

A total of 55 left-over stool samples were obtained from healthcare check-ups program at the Golden Jubilee Medical Center Mahidol University, Nakhon Pathom, Thailand, in 2022. Samples were cultured in MacConkey agar supplemented with 2 mg/L colistin. *Citrobacter* isolates were identified using traditional biochemical tests (Farmer et al., 1985) and species-level identification was confirmed by Biotyper (matrix-assisted laser desorption/ionization time-of-flight (MALDI-TOF) mass spectrometry) according to the manufacturer’s protocol (Bruker Daltonik, Leipzig, Germany). Colistin-resistant isolates were further confirmed by the gold standard broth-microdilution method defined by the Clinical and Laboratory Standards Institute (CLSI) (Clinical Laboratory and Standards Institute (CLSI), 2020). The presence of *mcr-*1 to *mcr*-10 was screened by multiplex PCR using the previously described protocols (Lescat et al., 2018; Wang et al., 2020; Borowiak et al., 2020), and the gene sequence was confirmed by Sanger DNA sequencing.

### Antimicrobial susceptibility testing (AST)

The minimum inhibitory concentrations (MICs) of amikacin, cefotaxime, ceftazidime, ciprofloxacin, chloramphenicol, colistin, gentamicin, imipenem, meropenem, nalidixic acid, tetracycline, and tigecycline were determined by broth microdilution method (BMD). MIC of fosfomycin was investigated by agar dilution method, which is recommended by CLSI. *Escherichia coli* ATCC 25922 was used as a quality control strain. The results were interpreted according to CLSI guideline.

### Whole−genome sequencing (WGS) and bioinformatics analysis

Genomic DNA (gDNA) of *C. amalonaticus* and *C. sedlakii* were extracted using PureLink^®^ Genomic DNA Kits (Invitrogen) according to the manufacturer’s instructions. The DNA samples were subsequently sequenced through NovaSeq 6000-PE150 platform (Illumina, San Diego, CA, USA) to generate paired-end 150-bp reads. The raw reads were then checked for quality and trimming using FastQC and TrimGalore, respectively (Andrews, 2022). *De novo* assembly was performed by SPAdes genome assembler version 3.15.3 (Prjibelski et al., 2020) to obtain contigs. The assembled contigs were then annotated through PROKKA and RAST server (Aziz et al., 2008; Seemann, 2014). Acquired antimicrobial resistant genes and plasmid replicons were determined using Resfinder (Bortolaia et al., 2020) and PlasmidFinder (Carattoli and Hasman, 2020), respectively. Additionally, the assembled contigs were also used for species identification using ribosomal multilocus sequence typing (rMLST). Based on the seven house-keeping genes (*aspC, clpX, fadD, mdh, arcA, dnaG and lysP*), the sequence type (ST) was identified using PubMLST server (Jolley and Maiden, 2010). Furthermore, the phylogenetic tree was performed and visualized through Roary (Page et al., 2015) and iTOL (Letunic and Bork, 2021), respectively.

### Plasmid characterization

Plasmid profiles of isolates containing *mcr* genes were characterized by pulsed-field gel electrophoresis with S1 nuclease (S1-PFGE) (Barton et al., 1995). Briefly, bacterial genomic DNA was embedded in plugs and digested with S1 nuclease (Fermentas, USA). Then, the linearized plasmid DNA was separated using a CHEF-DRIII system (Bio-Rad, Hercules, USA). *Salmonella braenderup* H9812 digested with *Xba*I was used as a reference DNA size marker. The location of the *mcr* gene in the plasmids was investigated by Southern blot analysis with a specific probe. The probe was labeled and hybridized using DIG-High Prime DNA Labeling and Detection Starter Kit II (Roche Diagnostics, Indianapolis, IN, USA) according to the manufacturer’s protocol. Transferability of plasmids harboring *mcr* gene was determined by plasmid conjugation experiment using the filter-mating technique as previously described (Khajanchi et al., 2019). Briefly, *Citrobacter* isolates harboring *mcr* gene and *Escherichia coli* J53, which is resistant to sodium azide were used as donors and recipients, respectively. The donor and recipient were mixed at a ratio of 1:2 on a filter and incubated on LB plate at 37°C for 4 hr. Transconjugants were selected on MacConkey agar containing 2 mg/L of colistin and 150 mg/L of sodium azide. Then, MALDI-TOF MS was used for identification of transconjugants and the presence of *mcr* gene was investigated by PCR to ensure that the plasmid was successfully transferred to the recipient strain.

### Nucleotide sequence accession numbers

The nucleotide sequences of pSY_CA01 and pSY_CS01 have been deposited in in the NCBI database with GenBank accession numbers JALNMK010000021 and JALNML010000026, respectively. The draft genomes of *C. amalonaticus* SY-CA35 and *C. sedlakii* SY-CS04 are also available in the NCBI database with accession numbers PRJNA827636 and PRJNA827638, respectively.

## Results

### Bacterial isolation and identification

**Figure 1:**
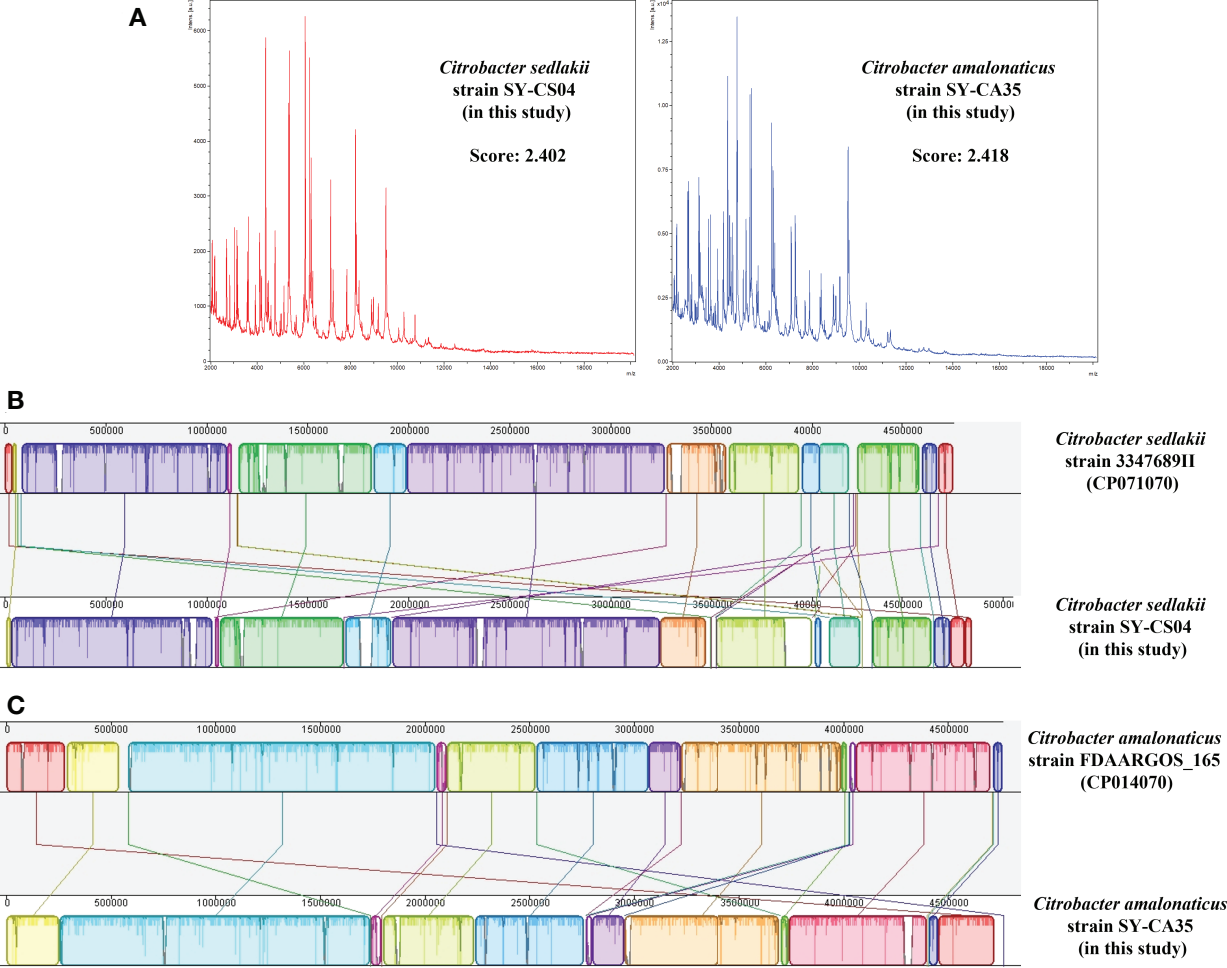
Identification of *Citrobacter* spp. **(A)** Mass fingerprinting of *C. sedlakii* and *C*. *amalonaticus* from Matrix-Assisted Laser Desorption/Ionization-Time Of Flight Mass Spectrometry (MALDI-TOF-MS). **(B)** Sequence alignment of *C. sedlakii* strain SY-CS04 with *C*. *sedlakii* strain 3347689II (accession no. CP071070). **(C)** Sequence alignment of *C*. *amalonaticus* strain SY-CA35 with *C*. *amalonaticus* strain FDAARGOS_165 (accession no. CP014070).

Based on MALDI-TOF MS experiment, two bacterial isolates, SY-CS04 and SY-CA35, were identified as *C. sedlakii* and *C. amalonaticus*, respectively (**Figure 1A**). This result is in an agreement with ribosomal multilocus sequence typing (rMLST), which showed that SY-CS04 and SY-CA35 were *C. sedlakii* and *C. amalonaticus*, respectively. Sequence alignment with reference strains showed that SY-CS04 and SY-CA35 had high sequence similarity to *C. sedlakii* (accession no. CP071070) and *C. amalonaticus* (accession no. CP014070), respectively (**Figures 1B, C**). Taken together, SY-CS04 and SY-CA35 have been identified as *C. sedlakii* and *C. amalonaticus*, respectively.

### Antimicrobial susceptibility testing and screening of *mcr* genes

Both SY-CS04 and SY-CA35 were susceptible to most of the antibiotics tested except for colistin (**Table 1**). SY-CA35 also exhibited resistance to nalidixic acid. The presence of *mcr* genes was sought by multiplex-PCR and the results showed that both isolates were positive for *mcr-3*. Then, the sequence of the gene was confirmed by Sanger DNA sequencing, which revealed that both isolates harbor *mcr-3.5* gene, with 100% identity to the reference sequence (accession number NG_055782.1).

**Table 1:** The minimum inhibitory concentrations (MICs) of bacterial isolates.

Isolate	Minimal Inhibitory Concentrations; MICs (mg/L)												
	AK	CTX	CAZ	CIP	C	CL	FOS	GM	IPM	MEM	NA	TE	TGC
SY-CS04	1	≤0.25	0.5	0.5	16	4	4	≤0.25	2	≤0.25	4	2	0.5
SY-CA35	1	≤0.25	0.5	0.5	16	8	4	≤0.25	2	≤0.25	32	2	0.5
*E. coli* J53	4	≤0.25	0.5	0.5	8	≤0.25	8	2	2	≤0.25	4	2	0.5
(T) SY-CS04	4	≤0.25	0.5	0.5	8	4	8	2	2	≤0.25	4	2	0.5
(T) SY-CA35	4	≤0.25	0.5	0.5	8	4	8	2	2	≤0.25	4	2	0.5

AK, amikacin; CTX, cefotaxime; CAZ, ceftazidime; CIP, ciprofloxacin; C, chloramphenicol; CL, colistin; FOS, fosfomycin; GM, gentamicin; IPM, imipenem; MEM, meropenem; NA, nalidixic acid; TE, tetracycline; TGC, tigecycline. The alphabet letter (T) represents the corresponding transconjugants.

### Genomic analysis of *Citrobacter* isolates

**Figure 2:**
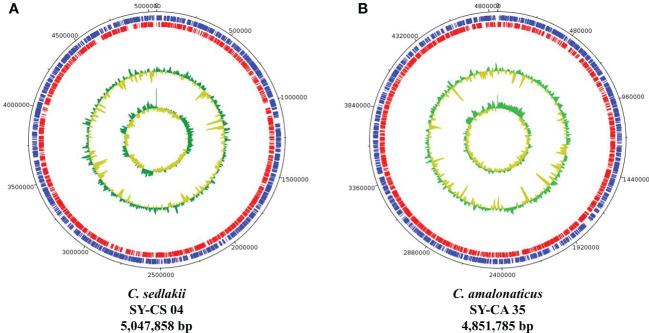
Overview of genomic structure of *Citrobacter* isolates. **(A)** *Citrobacter sedlakii* SY-CS04. **(B)** *Citrobacter amalonaticus* strain SY-CA35. The inner circle and outer circle represent GC skew and GC content, respectively. The protein-coding gene on forward strand and reverse strand represent in blue and red, respectively.

**Figure 3:**
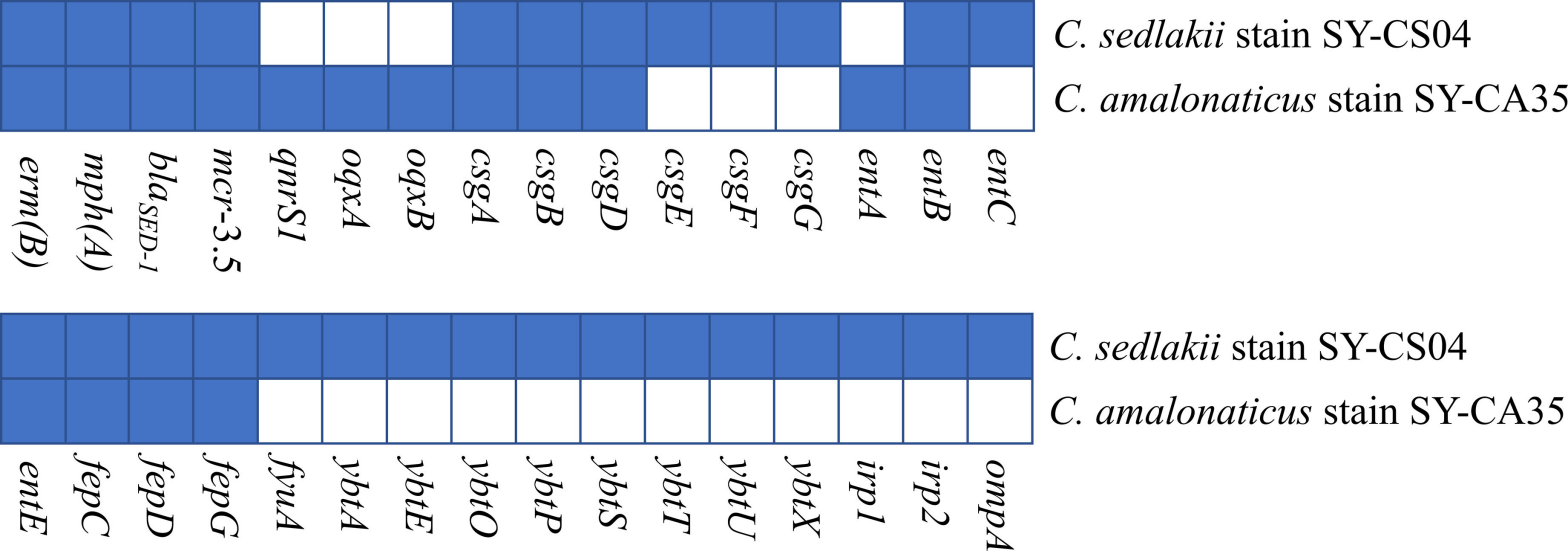
Antimicrobial resistance and virulence-associated profiles of the 2 *Citrobacter* isolates. Blue squares indicate the presence of genes while white squares represent the absence of genes.

**Figure 4:**
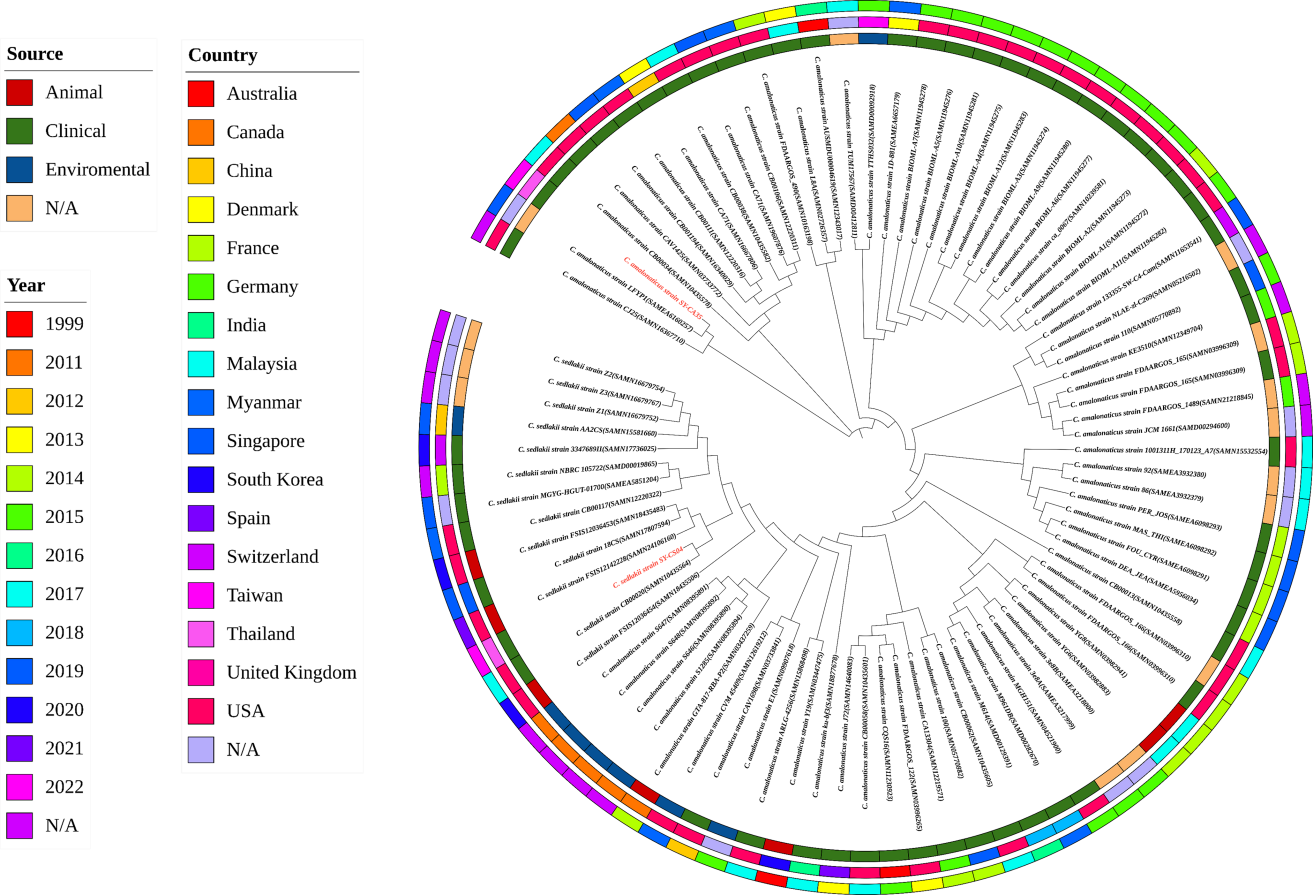
Roary matrix-based gene sequence analysis of 86 *Citrobacter* isolates. The source of the isolates is shown in the inner ring. The location of the isolates is depicted in the middle ring and the year of the isolates is indicated by the outer ring. Isolates in this study including SY-CS04 and SY-CA35 were colored in red.

As revealed by whole-genome sequencing, the genomic sizes of SY-CS04 and SY-CA35 were 5,047,858 and 4,851,785-bp, respectively. The GC content of SY-CS04 was 54.46% while that of SY-CA35 was 53.38% (**Figure 2**). Based on Resfinder analysis, the acquired resistant genes in both isolates were discovered (**Figure 3**). Both SY-CS04 and SY-CA35 possessed genes conferring resistance to macrolides (*erm(B)*, *mph(A)*), β-lactams (*bla_SED-1_*), and colistin (*mcr-3.5*). It is worth noting that these two isolates did not contain any other major antimicrobial resistant determinants found in multidrug resistant Enterobacterales. Additionally, SY-CA35 also carried quinolone-resistant gene (*qnrS1*) and fluoroquinolone-resistant genes (*oqxA*, *oqxB*). Virulence factors of SY-CS04 and SY-CA35 were predicted by VirulenceFinder. The presence of genes encoding extracellular nucleation factors (*csgA, csgB, csgD*), enterobactin (*entB, entE*), siderophores transportation (*fepC, fepD, fepG*) were found in both isolates. SY-CA35 also carried enterobactin (*entA*) while SY-CS04 carried enterobactin (*entC*). Furthermore, SY-CS04 also contained genes encoding extracellular nucleation factors (*csgE, csgG, csgF*), yersiniabactin receptor (*fyuA*), siderophore yersiniabactin (*ybtA, ybtE, ybtO, ybtP, ybtS, ybtT, ybtU, ybtX*), iron regulatory proteins (*irp1, irp2*), and outer membrane protein A (*ompA*). MLST analysis performed by PubMLST revealed that the sequence of SY-CS04 and SY-CA35 did not match with the existed sequences in the database. Therefore, SY-CS04 and SY-CA35 were newly assigned as ST682 and ST681, respectively (**Table 2**). Then phylogenetic tree was generated through roary bacterial genome analysis. All available genome data of *C. sedlakii* and *C. amalonaticus* were retrieved from NCBI genome database. Roary matrix-based gene sequence analysis generated a pangenome consisting of 37,961 gene clusters of 86 whole genomes (**Figure 4**). The tree revealed that SY-CS04 and SY-CA35 were closely related to a clinical isolate *C. sedlakii* stain CB00020 (accession no. SAMN10435564) and a clinical isolate *C. amalonaticus* stain LFYP1 (accession no. SAMEA6160257) from the USA, respectively.

**Table 2:** Genomic and plasmid profiles of *Citrobacter* isolates.

Strain	Allelic profiles of house-keeping loci							Sequence type (ST)	Plasmid size (kb)	Inc group	Transfer rates
	aspC	clpX	fadD	mdh	arcA	dnaG	lysP				
*Citrobacter* *sedlakii* strain SY-CS04	215	253	274	205	133	196	236	682	~78.2	IncFII (pCoo)	4.6×10^-4^
									~100	IncFIIs/ IncR	ND
*Citrobacter* *amalonaticuus* strain SY-CA35	137	152	214	213	75	184	186	681	~78.2	IncFII (pCoo)	1.03×10^-4^
									~33.3	IncFII (pMET)	ND

ND, not determined.

### Plasmid characterization

**Figure 5:**
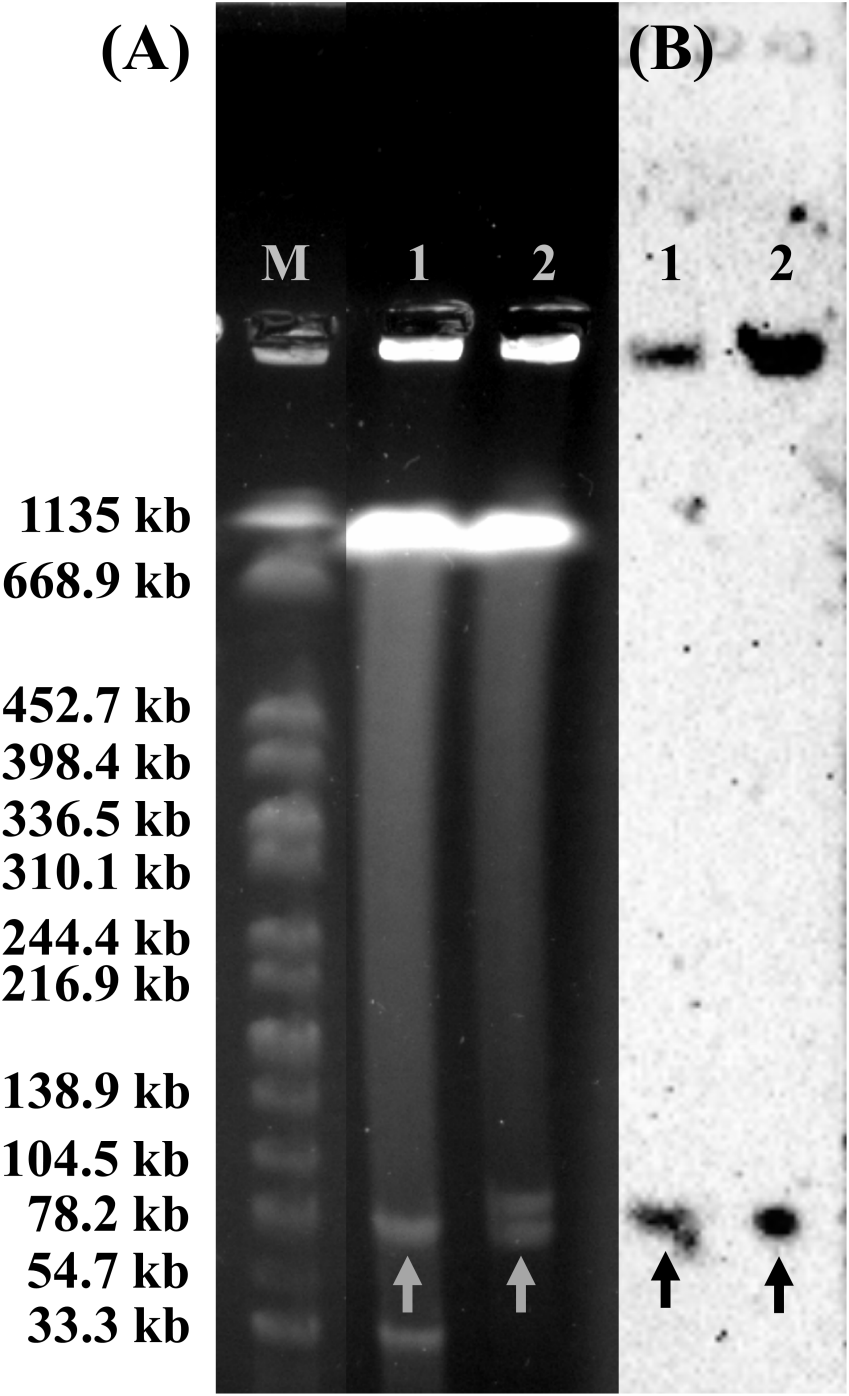
Plasmid profile analysis of *Citrobacter* isolates harboring *mcr-3.5* gene by S1-PFGE and DNA hybridization. **(A)** The profile of total DNA treated with S1 nuclease and **(B)** relative hybridization of *mcr-3* probe. Lane M, molecular standard, which is *Salmonella braenderup* H9812 digested with *Xba*I. Lane 1, *Citrobacter amalonaticus* strain SY-CA35. Lane 2, *Citrobacter sedlakii* strain SY-CS04. Arrows indicate the locations of plasmid harboring *mcr-3* gene.

The plasmid profiles of SY-CS04 and SY-CA35 were characterized by S1-PFGE (**Figure 5A**), which revealed the presence of two plasmids in each of the two strains. In SY-CS04, the plasmid sizes were ~78.2 and ~100 kb, while in SY-CA35, the plasmid sizes were ~33.3 and ~78.2 kb. The location of *mcr-3.5* gene was then identified using DNA hybridization with a specific probe (**Figure 5B**), which revealed that the gene was located on the ~78.2 kb plasmid in both SY-CS04 and SY-CA35. The incompatibility group of the plasmids was identified through PlasmidFinder. IncFII(pCoo) plasmid was found in both SY-CS04 and SY-CA35. In addition, an IncFII(S)/IncR plasmid was found in SY-CS04 while an IncFII(pMET) plasmid was found in SY-CA35 (**Table 2**). In combination with S1-PFGE, these results suggest that the *mcr-3.5* gene is located on IncFII(pCoo) plasmid with a size of ~78.2 kb in both strains. IncFII(pMET) is the plasmid with a size of ~33.3 kb in SY-CA35. IncFII(S)/IncR plasmid is a hybrid plasmid with the size of ~100 kb in SY-CS04.

**Figure 6:**
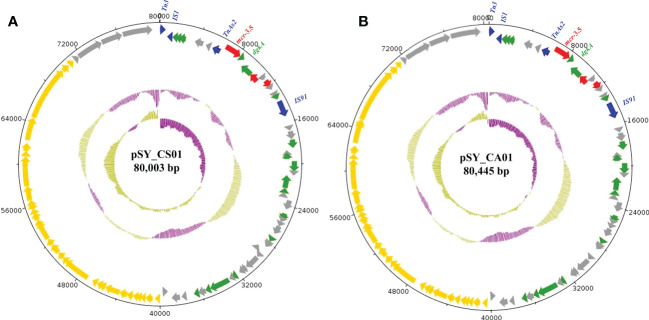
Structure of the IncFII(pCoo) harboring *mcr-3.5*, including pSY_CS01 **(A)** and pSY_CA01 **(B)**. The inner circle and outer circle represent GC skew and GC content, respectively. The arrows indicate the directions of gene transcription. The red arrows represent antimicrobial resistance genes, the green arrows show other functional genes, the blue arrows show mobile element-encoding genes, the yellow arrows show IncF plasmid conjugative element and grey arrows for hypothetical protein-encoding genes.

**Figure 7:**
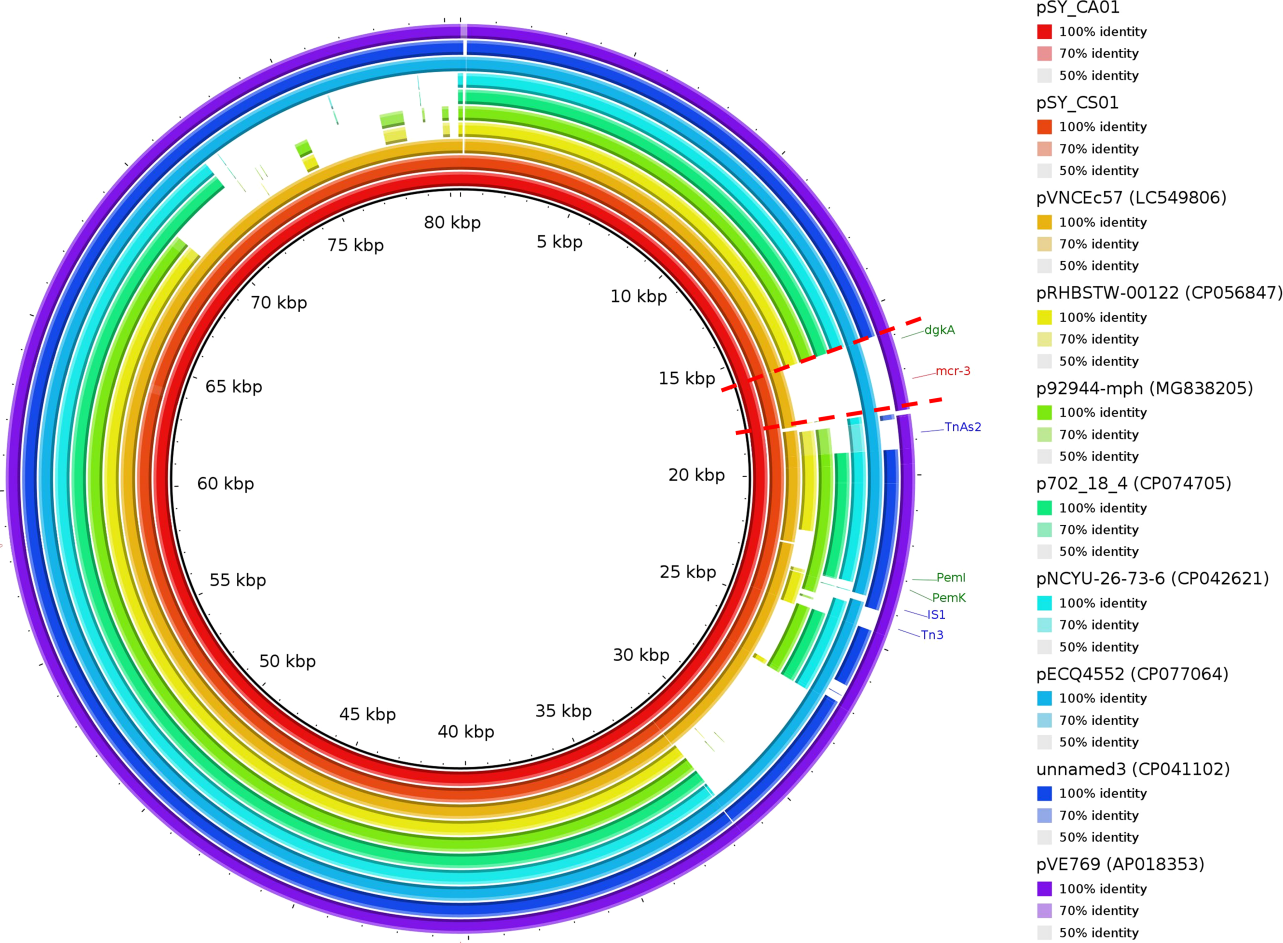
Circular comparison of IncFII(pCoo) harboring *mcr-3.5*, pSY_CS01 and pSY_CA01 with eight homologous plasmids with considerable query coverage.

**Figure 8:**
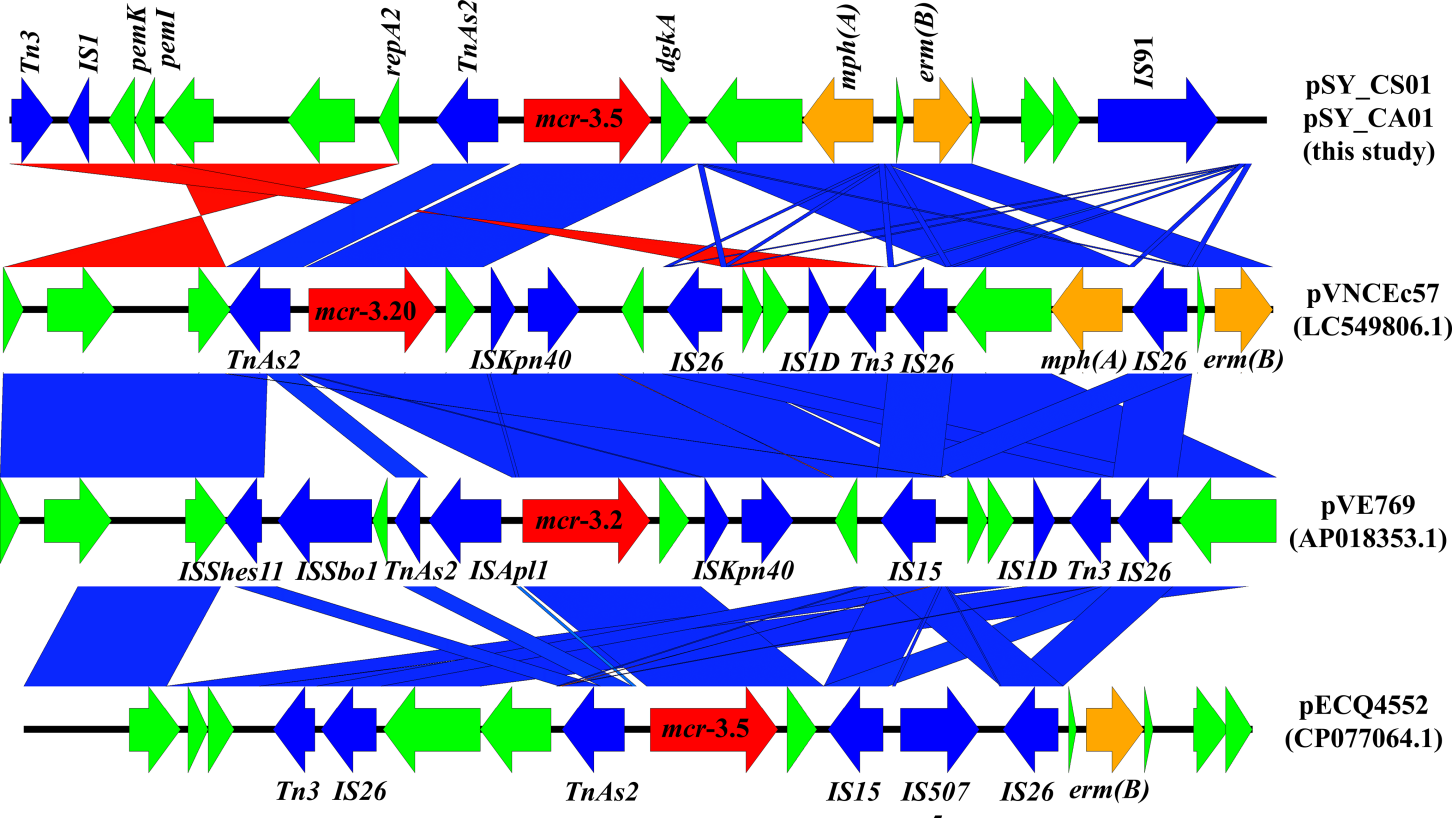
Linear comparison of surrounding regions of *mcr-3*. The arrows indicate directions of gene transcription. *mcr-3* is labeled in red arrow, while other antimicrobial resistance genes are labeled in orange. Mobile genetic elements are indicated in blue and other functional gene are in green.

**Figure 9:**
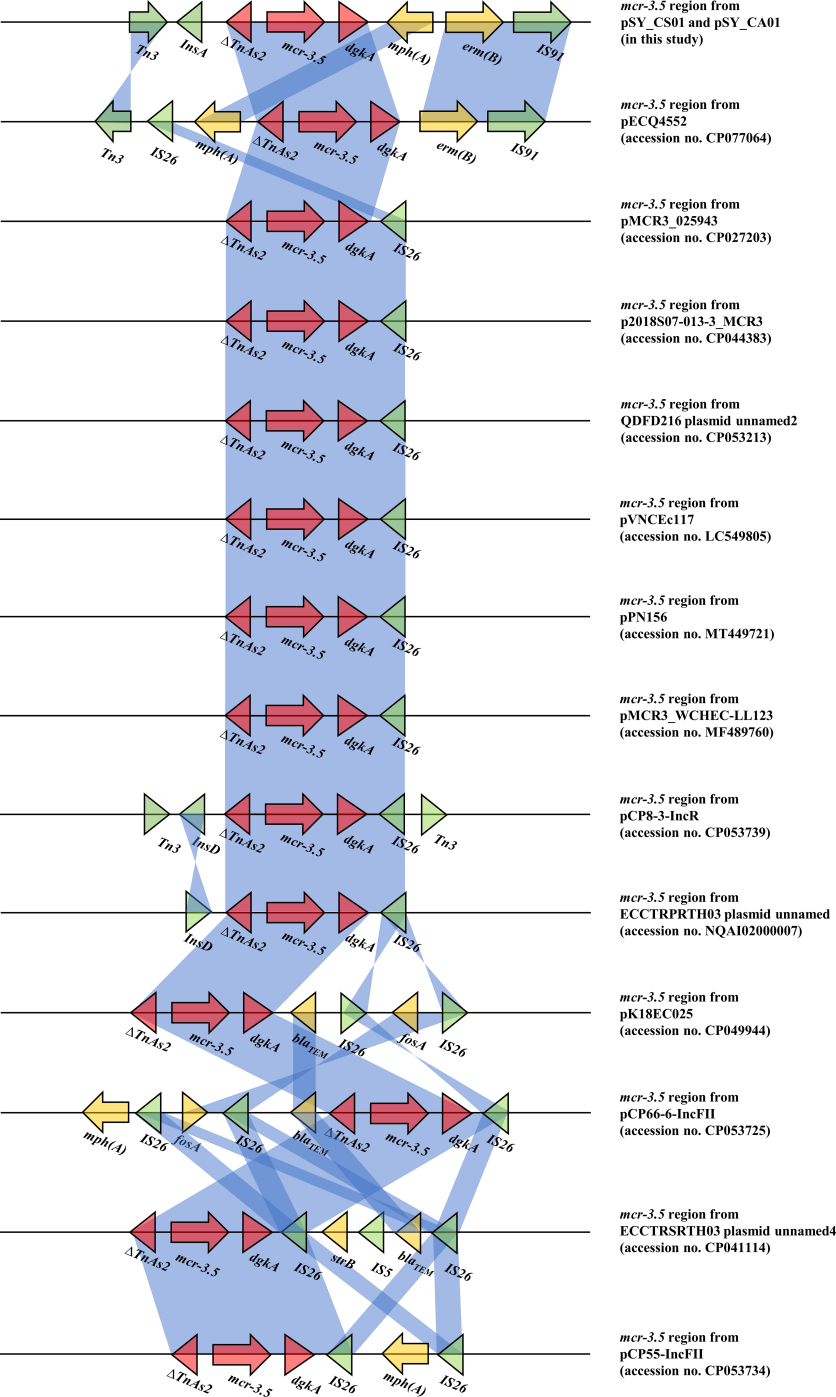
Comparison of 14 *mcr-3.5* regions from 16 plasmids. The arrows indicate directions of gene transcription. Shading in light blue denotes regions of homology (nucleotide identity 95%).

Bioinformatic analysis revealed that *mcr*-3.5 was located on a plasmid of SY-CS04 and SY-CA35, which were then designated as pSY_CS01 and pSY_CA01, respectively. The size of pSY_CS01 and pSY_CA01 were 80,003-bp with 52.59% GC content and 80,445-bp with 52.67% GC content, respectively. Both plasmids belonged to IncFII(pCoo) plasmid replicon type and contained 279 predicted ORFs encoding proteins with over 50 amino acids long (**Figure 6**). Notably, the genetic environment of *mcr-3.5* in pSY_CS01 and pSY_CA01 was the same, which is Tn*3-*IS*1-*ΔTnAs*2-mcr3.5-dgkA-*IS*91*. Plasmids pSY_CS01 and pSY_CA01 were then blasted through BLASTN and 8 best matches with query cover >75% and identity >99% were identified, these include pVNCEc57 (LC549806.1), pRHBSTW-00122 (CP056847.1), p92944-mph (MG838205.1), p702_18_4 (CP074705.1), pNCYU-26-73-6 (CP042621.1), pECQ4552 (CP077064.1), unnamed3 (CP041102.1), and pVE769 (AP018353.1). In this regard, *mcr*-3 was identified in only 3 plasmids, which were found in *E. coli* including pVNCEc57 from Vietnam, pECQ4552 from France, and pVE769 from Vietnam (**Figure 7**). Then, the sequences of plasmid containing *mcr-3* were compared with sequence from our study. As shown in **Figures 7**, **8**, all plasmids shared the same backbone region. However, the surrounding region of *mcr-3* from our study was different from the sequences in the database suggesting that insertion of genetic elements had occurred. Moreover, mobile genetic element also contained toxin/antitoxin system indicating the stabilization of mobile genetic element within plasmid (Song and Wood, 2020). In addition, the surrounding region of *mcr-3.5* in this study were compared with 13 plasmids harboring *mcr-3.5* (**Figure 9**), which were retrieved from NCBI database. The result showed that ΔTnAs*2*-*mcr-3.5*-*dgkA* region were found in all sequences. Various insertion sequences (IS) such as IS*91*, Tn*3*, IS*26* were also identified at the upstream or downstream of that region.

Furthermore, the transferability of plasmids harboring *mcr-3.5* gene was determined by plasmid conjugation assay. Both plasmids were successfully transferred to *E. coli* J53 with high transferring efficiency, ranging from 1.03×10^-4^ - 4.6×10^-4^ colony forming units (CFU) per recipient cell. Both transconjugants exhibited a 16-fold (4 mg/L) increase in the colistin MICs when compared with that of the recipient cell (*E. coli* J53) (**Table 1**). These results suggested that the *mcr-3.5* gene on IncFII(pCoo) plasmid can be transferred and expressed in transconjugants.

## Discussion

The *mcr-3* gene was first reported by Yin W et al. in China (Yin et al., 2017). The gene was located on IncHI2 replicon type plasmid found in *E. coli* isolated from pig. Currently, more than 40 variants of *mcr-3* have been deposited in the NCBI database, indicating that the *mcr-3* gene is widespread and genetically diverse. In addition, the gene has been reported to be associated with three replicon types including IncP1, IncFII and IncI1, which can be found in various species of bacteria including *Aeromonas* spp., *E. coli*, *K. pneumoniae*, *Salmonella*, and *Enterobacter* spp. *Citrobacter* spp. are opportunistic bacterial pathogens that can cause both hospital- and community-acquired infections. It has been reported that *Citrobacter* spp. represent up to 6% of all isolated Enterobacterales from clinical specimens (Oberhettinger et al., 2020). In this study, we identified and characterized two clinical isolates of *Citrobacter* spp. (SY-CS04 and SY-CA35) harboring *mcr-3.5*. Identification of these isolates at species level was not possible with biochemical tests. Yet, it has been reported that 16S rRNA sequences displays limited resolution distinguishing only three groups within the genus (Clermont et al., 2015). Therefore, in our study, MALDI-TOF MS has been used for identification and the results yielded a category A identification (score > 2.0), which can be considered a reliable identification. In addition, rMLST, an approach of integrating taxonomy and typing of microbial communities by analyzing variation in 53 genes encoding ribosome protein subunits (rps genes) has been used to confirm the species and the results were in an agreement with MALDI-TOF MS, which identified SY-CS04 and SY-CA35 as *C. sedlakii* and *C. amalonaticus*, respectively. For *mcr*-*3.5*, it was first identified on IncP1 plasmid found in *E. coli* in China (Liu et al., 2017). It has also been found in other plasmid replicon types including IncR, IncFII, and IncFII(pCoo). In our study, IncFII(pCoo) harboring *mcr*-3.5, namely pSY_CS01 and pSY_CA01 were identified in *C. sedlakii* SY-CS04 and *C. amalonaticus* SY-CA35, respectively. It was noted that the genetic context of pSY_CS01 and pSY_CA01 were the same. Since both *Citrobacter* isolates were from the same human subject, the two plasmids might be derived from the same clone. Comparison of 16 *mcr*-*3.5* loci showed that the genetic context of ΔTnAs*2*-*mcr-3.5*-*dgkA* might be the conserved structure of the *mcr*-*3.5* locus. Interestingly, this genetic context has been interrupted by various IS elements at the upstream or downstream, suggesting that the area surrounding this conserved region could be the high-frequency region for insertion of mobile genetic elements. IncFII type is a low-copy number plasmid. It is one of the narrow-host range plasmids that are commonly found in *E. coli* (Carattoli, 2009). However, IncFII plasmid can disseminate and replicate in a variety of Enterobacterales, which contributes a crucial role for spreading of antimicrobial resistant genes (Chen et al., 2014). As shown in **Figures 7**, **8**, comparison of pSY_CS01 and pSY_CA01 with plasmids containing *mcr-3* from *E. coli* recovered from Vietnam and France showed that these plasmids share a similar backbone. Since these plasmids have been recovered from different species and geographical locations, these results suggest that pSY_CS01 and pSY_CA01 may contribute to the transmission of *mcr-3.5* among other Enterobacterales species.

There is an evidence that the presence of *mcr* genes in food animals significantly increased the risk of direct contact with bacteria harboring *mcr* genes, in particular transmission of Enterobacterales to humans (Liu et al., 2016; Trung et al., 2017; Shen et al., 2018). In addition, several research groups have proposed the other risk factors with high potential for dissemination of *mcr* genes to humans, especially environmental contaminations (Liu et al., 2016; Malhotra-Kumar et al., 2016; Trung et al., 2017; Shen et al., 2018; Agnoletti et al., 2018). Based on a meta-analysis of publications in six major databases published between 18 November 2015 and 30 December 2018, environmental samples exhibited the highest cumulative average prevalence of *mcr* genes, followed by animals, food, and humans. In human, 62% were from clinical patients and 38% were from asymptomatic carriers (Elbediwi et al., 2019). Thus, based on these findings, the presence of *Citrobacter* spp. carrying *mcr gene* in healthy individual found in our study may be due to ingestion of contaminated food animals or environmental. Therefore, strategic action plans, such as surveillance programs of human, animal and environmental setting which is the perspective of “One Health” to control and prevent the spread of *mcr* genes are urgently needed.

In conclusion, in this study, two colistin-resistant *Citrobacter* spp. were isolated from feces of healthy individuals. The two isolates, *C. sedlakii* strain SY-CS04 and *C. amalonaticus* strain SY-CA35 were newly assigned to ST682, and ST681, respectively. Both isolates exhibited resistant phenotype only to colistin, which is mediated by IncFII(pCoo) plasmid harboring *mcr-3.5*. These plasmids displayed high transferring efficiency and conferred colistin resistance to transconjugant *E. coli*. These findings suggest the widespread of *mcr* plasmid-mediated colistin resistance among Enterobacterales species. It is worth noting that both *Citrobacter* isolates harbored only *mcr* gene but no any other major antimicrobial resistant determinants found in multidrug resistant Enterobacterales. To the best of our knowledge, this is the first report of *mcr* alleles in *C*. *sedlakii* and *mcr-3* in *C. amalonaticus*. Due to the fact that the two *Citrobacter* spp. were isolated from the healthy individual and lacked major resistant determinants in multidrug resistant Enterobacterales, our results suggested an ongoing evolution of *mcr* gene in human under unknown selection. More importantly, since *Citrobacter* spp. is one of the most abundant intestinal bacteria, our findings supported the theory that *Citrobacter* may serve as a reservoir of antibiotic resistant genes, which poses a significant public health threat.

## Data availability statement

The datasets presented in this study can be found in online repositories. The names of the repository/repositories and accession number(s) can be found in the article/supplementary material.

## Ethics statement

Ethical approval in this study was waived by the Mahidol University Central Institutional Review Board (MU-CIRB), Mahidol University (Nakhon Pathom, Thailand) because the sample used is anonymous. All protocols were in accordance with the ethical standards of our institution and with the 1964 Helsinki Declaration and its later amendments or comparable ethical standards.

## Author contributions

SY conceived the project proposal. TP isolated and identified the bacteria and performed antibiotic susceptibility testing. TP and NS performed bioinformatics analysis of WGS. TP and SW performed molecular experiments including PCR and PFGE. SY, ST, and CI-N-A evaluated the data and provided expertise and feedback. TP wrote the preliminary draft of the manuscript. SY edited and finalized the manuscript. All authors contributed to the article and approved the submitted version.

## References

[B1] AbuOun M. Stubberfield E. J. Duggett N. A. Kirchner M. Dormer L. Nunez-Garcia J.. (2018). Mcr-1 and mcr-2 (mcr-6.1) variant genes identified in moraxella species isolated from pigs in great Britain from 2014 to 2015. J. Antimicrob. Chemother. 73, 2904. doi: 10.1093/jac/dky272 30053008 PMC6148207

[B2] Agnoletti F. Brunetta R. Bano L. Drigo I. Mazzolini E. (2018). Longitudinal study on antimicrobial consumption and resistance in rabbit farming. Int. J. Antimicrob. Agents 51, 197–205. doi: 10.1016/j.ijantimicag.2017.10.007 29111433

[B3] Andrews S. FastQC: A quality control tool for high throughput sequence data. Available at: http://www.bioinformatics.babraham.ac.uk/projects/fastqc/ (Accessed February 2, 2022).

[B4] Aziz R. K. Bartels D. Best A. A. DeJongh M. Disz T. Edwards R. A.. (2008). The RAST server: rapid annotations using subsystems technology. BMC Genomics 9, 75. doi: 10.1186/1471-2164-9-75 18261238 PMC2265698

[B5] Barton B. M. Harding G. P. Zuccarelli A. J. (1995). A general method for detecting and sizing large plasmids. Anal. Biochem. 226, 235–240. doi: 10.1006/abio.1995.1220 7793624

[B6] Bitar I. Papagiannitsis C. C. Kraftova L. Chudejova K. Mattioni Marchetti V. Hrabak J. (2020). Detection of five mcr-9-Carrying enterobacterales isolates in four Czech hospitals. mSphere 5, e01008-20. doi: 10.1128/mSphere.01008-20 33298573 PMC7729258

[B7] Borowiak M. Baumann B. Fischer J. Thomas K. Deneke C. Hammerl J. A.. (2020). Development of a novel mcr-6 to mcr-9 multiplex PCR and assessment of mcr-1 to mcr-9 occurrence in colistin-resistant salmonella enterica isolates from environment, feed, animals and food, (2011-2018) in Germany. Front. Microbiol. 11. doi: 10.3389/fmicb.2020.00080 PMC7011100 32117115

[B8] Borowiak M. Fischer J. Hammerl J. A. Hendriksen R. S. Szabo I. Malorny B. (2017). Identification of a novel transposon-associated phosphoethanolamine transferase gene, mcr-5, conferring colistin resistance in d-tartrate fermenting salmonella enterica subsp. enterica serovar paratyphi b. J. Antimicrob. Chemother. 72, 3317–3324. doi: 10.1093/jac/dkx327 28962028

[B9] Bortolaia V. Kaas R. S. Ruppe E. Roberts M. C. Schwarz S. Cattoir V.. (2020). ResFinder 4.0 for predictions of phenotypes from genotypes. J. Antimicrob. Chemother. 75, 3491–3500. doi: 10.1093/jac/dkaa345 32780112 PMC7662176

[B10] Carattoli A. (2009). Resistance plasmid families in enterobacteriaceae. Antimicrob. Agents Chemother. 53, 2227–2238. doi: 10.1128/AAC.01707-08 19307361 PMC2687249

[B11] Carattoli A. Hasman H. (2020). PlasmidFinder and *in silico* pMLST: Identification and typing of plasmid replicons in whole-genome sequencing (WGS). Methods Mol. Biol. 2075, 285–294. doi: 10.1007/978-1-4939-9877-7_20 31584170

[B12] Chen L. Mathema B. Chavda K. D. DeLeo F. R. Bonomo R. A. Kreiswirth B. N. (2014). Carbapenemase-producing klebsiella pneumoniae: molecular and genetic decoding. Trends Microbiol. 22, 686–696. doi: 10.1016/j.tim.2014.09.003 25304194 PMC4365952

[B13] Clermont D. Motreff L. Passet V. Fernandez J.-C. Bizet C. Brisse S. (2015). Multilocus sequence analysis of the genus citrobacter and description of citrobacter pasteurii sp. nov. Int. J. Syst. Evol. Microbiol. 65, 1486–1490. doi: 10.1099/ijs.0.000122 25687346

[B14] Clinical Laboratory and Standards Institute (CLSI) (2020). Performance standards for antimicrobial susceptibility testing. 32nd ed (Wayne, PA, USA: Clinical and Laboratory Standards Institute). M100 2022.

[B15] Eiamphungporn W. Yainoy S. Jumderm C. Tan-Arsuwongkul R. Tiengrim S. Thamlikitkul V. (2018). Prevalence of the colistin resistance gene mcr-1 in colistin-resistant escherichia coli and klebsiella pneumoniae isolated from humans in Thailand. J. Glob. Antimicrob. Resist. 15, 32–35. doi: 10.1016/j.jgar.2018.06.007 29935331

[B16] Elbediwi M. Li Y. Paudyal N. Pan H. Li X. Xie S.. (2019). Global burden of colistin-resistant bacteria: Mobilized colistin resistance genes study, (1980-2018). Microorganisms 7, 461. doi: 10.3390/microorganisms7100461 31623244 PMC6843232

[B17] Faccone D. Albornoz E. Tijet N. Biondi E. Gomez S. Pasterán F.. (2019). Characterization of a multidrug resistant citrobacter amalonaticus clinical isolate harboring blaNDM-1 and mcr-1.5 genes. Infect. Genet. Evol. 67, 51–54. doi: 10.1016/j.meegid.2018.10.020 30389546

[B18] Farmer J. J. Davis B. R. Hickman-Brenner F. W. McWhorter A. Huntley-Carter G. P. Asbury M. A.. (1985). Biochemical identification of new species and biogroups of enterobacteriaceae isolated from clinical specimens. J. Clin. Microbiol. 21, 46–76. doi: 10.1128/jcm.21.1.46-76.1985 3881471 PMC271578

[B19] Hanson N. D. Sanders C. C. (1999). Regulation of inducible AmpC beta-lactamase expression among enterobacteriaceae. Curr. Pharm. Des. 5, 881–894. 10539994

[B20] Hu Y.-Y. Wang Y.-L. Sun Q.-L. Huang Z.-X. Wang H.-Y. Zhang R.. (2017). Colistin resistance gene mcr-1 in gut flora of children. Int. J. Antimicrob. Agents 50, 593–597. doi: 10.1016/j.ijantimicag.2017.06.011 28668691

[B21] Jacobson K. L. Cohen S. H. Inciardi J. F. King J. H. Lippert W. E. Iglesias T.. (1995). The relationship between antecedent antibiotic use and resistance to extended-spectrum cephalosporins in group I beta-lactamase-producing organisms. Clin. Infect. Dis. 21, 1107–1113. doi: 10.1093/clinids/21.5.1107 8589129

[B22] Jiang X. Cui X. Liu W. Xu H. Zheng B. (2019). Genetic characterization of a novel sequence type of multidrug-resistant citrobacter freundii strain recovered from wastewater treatment plant. Infect. Drug Resist. 12, 2775–2779. doi: 10.2147/IDR.S213525 31564927 PMC6735533

[B23] Jolley K. A. Maiden M. C. J. (2010). BIGSdb: Scalable analysis of bacterial genome variation at the population level. BMC Bioinf. 11, 595. doi: 10.1186/1471-2105-11-595 PMC3004885 21143983

[B24] Kanamori H. Yano H. Hirakata Y. Endo S. Arai K. Ogawa M.. (2011). High prevalence of extended-spectrum β-lactamases and qnr determinants in citrobacter species from Japan: dissemination of CTX-M-2. J. Antimicrob. Chemother. 66, 2255–2262. doi: 10.1093/jac/dkr283 21733965

[B25] Khajanchi B. K. Kaldhone P. R. Foley S. L. (2019). Protocols of conjugative plasmid transfer in salmonella: plate, broth, and filter mating approaches. Methods Mol. Biol. 2016, 129–139. doi: 10.1007/978-1-4939-9570-7_12 31197715

[B26] Kieffer N. Royer G. Decousser J.-W. Bourrel A.-S. Palmieri M. Ortiz de la Rosa J.-M.. (2019). Mcr-9, an inducible gene encoding an acquired phosphoethanolamine transferase in escherichia coli, and its origin. Antimicrob. Agents Chemother. 63, e00965-19. doi: 10.1128/AAC.00965-19 31209009 PMC6709461

[B27] Lee C.-H. Lee Y.-T. Kung C.-H. Ku W.-W. Kuo S.-C. Chen T.-L.. (2015). Risk factors of community-onset urinary tract infections caused by plasmid-mediated AmpC β-lactamase-producing enterobacteriaceae. J. Microbiol. Immunol. Infect. 48, 269–275. doi: 10.1016/j.jmii.2013.08.010 24239065

[B28] Lescat M. Poirel L. Nordmann P. (2018). Rapid multiplex polymerase chain reaction for detection of mcr-1 to mcr-5 genes. Diagn. Microbiol. Infect. Dis. 92, 267–269. doi: 10.1016/j.diagmicrobio.2018.04.010 30220493

[B29] Letunic I. Bork P. (2021). Interactive tree of life (iTOL) v5: an online tool for phylogenetic tree display and annotation. Nucleic Acids Res. 49, W293–W296. doi: 10.1093/nar/gkab301 33885785 PMC8265157

[B30] Li X.-P. Fang L.-X. Jiang P. Pan D. Xia J. Liao X.-P.. (2017). Emergence of the colistin resistance gene mcr-1 in citrobacter freundii. Int. J. Antimicrob. Agents 49, 786–787. doi: 10.1016/j.ijantimicag.2017.04.004 28433744

[B31] Liu L. Feng Y. Zhang X. McNally A. Zong Z. (2017). New variant of mcr-3 in an extensively drug-resistant escherichia coli clinical isolate carrying mcr-1 and blaNDM-5. Antimicrob. Agents Chemother. 61, e01757-17. doi: 10.1128/AAC.01757-17 PMC5700326 28971871

[B32] Liu Y.-Y. Wang Y. Walsh T. R. Yi L.-X. Zhang R. Spencer J.. (2016). Emergence of plasmid-mediated colistin resistance mechanism MCR-1 in animals and human beings in China: a microbiological and molecular biological study. Lancet Infect. Dis. 16, 161–168. doi: 10.1016/S1473-3099(15)00424-7 26603172

[B33] Liu L.-H. Wang N.-Y. Wu A. Y.-J. Lin C.-C. Lee C.-M. Liu C.-P. (2018b). Citrobacter freundii bacteremia: Risk factors of mortality and prevalence of resistance genes. J. Microbiol. Immunol. Infect. 51, 565–572. doi: 10.1016/j.jmii.2016.08.016 28711438

[B34] Liu J. Yang Y. Li Y. Liu D. Tuo H. Wang H.. (2018a). Isolation of an IncP-1 plasmid harbouring mcr-1 from a chicken isolate of citrobacter braakii in China. Int. J. Antimicrob. Agents 51, 936–940. doi: 10.1016/j.ijantimicag.2017.12.030 29305957

[B35] Madec J. Y. Haenni M. Nordmann P. Poirel L. (2017). Extended-spectrum β-lactamase/AmpC- and carbapenemase-producing enterobacteriaceae in animals: a threat for humans? Clin. Microbiol. Infect. 23, 826–833. doi: 10.1016/j.cmi.2017.01.013 28143782

[B36] Malhotra-Kumar S. Xavier B. B. Das A. J. Lammens C. Hoang H. T. T. Pham N. T.. (2016). Colistin-resistant escherichia coli harbouring mcr-1 isolated from food animals in Hanoi, Vietnam. Lancet Infect. Dis. 16, 286–287. doi: 10.1016/S1473-3099(16)00014-1 26774248

[B37] Mmatli M. Mbelle N. M. Osei Sekyere J. (2022). Global epidemiology, genetic environment, risk factors and therapeutic prospects of mcr genes: A current and emerging update. Front. Cell. Infect. Microbiol. 12. doi: 10.3389/fcimb.2022.941358 PMC9462459 36093193

[B38] Mohanty S. Singhal R. Sood S. Dhawan B. Kapil A. Das B. K. (2007). Citrobacter infections in a tertiary care hospital in northern India. J. Infect. 54, 58–64. doi: 10.1016/j.jinf.2006.01.015 16815552

[B39] Oberhettinger P. Schüle L. Marschal M. Bezdan D. Ossowski S. Dörfel D.. (2020). Description of citrobacter cronae sp. nov., isolated from human rectal swabs and stool samples. Int. J. Syst. Evol. Microbiol. 70, 2998–3003. doi: 10.1099/ijsem.0.004100 32375941 PMC7395625

[B40] Ouchar Mahamat O. Kempf M. Lounnas M. Tidjani A. Hide M. Benavides J. A.. (2021). Epidemiology and prevalence of extended-spectrum β-lactamase- and carbapenemase-producing enterobacteriaceae in humans, animals and the environment in West and central Africa. Int. J. Antimicrob. Agents 57, 106203. doi: 10.1016/j.ijantimicag.2020.106203 33075511

[B41] Page A. J. Cummins C. A. Hunt M. Wong V. K. Reuter S. Holden M. T. G.. (2015). Roary: rapid large-scale prokaryote pan genome analysis. Bioinformatics 31, 3691–3693. doi: 10.1093/bioinformatics/btv421 26198102 PMC4817141

[B42] Papp-Wallace K. M. Endimiani A. Taracila M. A. Bonomo R. A. (2011). Carbapenems: past, present, and future. Antimicrob. Agents Chemother. 55, 4943–4960. doi: 10.1128/AAC.00296-11 21859938 PMC3195018

[B43] Phuadraksa T. Wichit S. Arikit S. Songtawee N. Yainoy S. (2022). Co-Occurrence of mcr-2 and mcr-3 genes on chromosome of multidrug-resistant escherichia coli isolated from healthy individuals in Thailand. Int. J. Antimicrob. Agents 60, 106662. doi: 10.1016/j.ijantimicag.2022.106662 36007781

[B44] Prjibelski A. Antipov D. Meleshko D. Lapidus A. Korobeynikov A. (2020). Using SPAdes *de novo* assembler. Curr. Protoc. Bioinf. 70, e102. doi: 10.1002/cpbi.102 32559359

[B45] Rahal J. J. (2008). The role of carbapenems in initial therapy for serious gram-negative infections. Crit. Care 12 Suppl 4, S5. doi: 10.1186/cc6821 PMC2391262 18495062

[B46] Sadek M. Ortiz de la Rosa J. M. Abdelfattah Maky M. Korashe Dandrawy M. Nordmann P. Poirel L. (2021). Genomic features of MCR-1 and extended-spectrum β-Lactamase-Producing enterobacterales from retail raw chicken in Egypt. Microorganisms 9, 195. doi: 10.3390/microorganisms9010195 33477851 PMC7832903

[B47] Samonis G. Karageorgopoulos D. E. Kofteridis D. P. Matthaiou D. K. Sidiropoulou V. Maraki S.. (2009). Citrobacter infections in a general hospital: characteristics and outcomes. Eur. J. Clin. Microbiol. Infect. Dis. 28, 61–68. doi: 10.1007/s10096-008-0598-z 18682995

[B48] Seemann T. (2014). Prokka: rapid prokaryotic genome annotation. Bioinformatics 30, 2068–2069. doi: 10.1093/bioinformatics/btu153 24642063

[B49] Sennati S. Di Pilato V. Riccobono E. Di Maggio T. Villagran A. L. Pallecchi L.. (2017). Citrobacter braakii carrying plasmid-borne mcr-1 colistin resistance gene from ready-to-eat food from a market in the chaco region of Bolivia. J. Antimicrob. Chemother. 72, 2127–2129. doi: 10.1093/jac/dkx078 28333311

[B50] Shahid M. (2010). Citrobacter spp. simultaneously harboring blaCTX-m, blaTEM, blaSHV, blaampC, and insertion sequences IS26 and orf513: an evolutionary phenomenon of recent concern for antibiotic resistance. J. Clin. Microbiol. 48, 1833–1838. doi: 10.1128/JCM.01467-09 20220171 PMC2863875

[B51] Shen Y. Zhou H. Xu J. Wang Y. Zhang Q. Walsh T. R.. (2018). Anthropogenic and environmental factors associated with high incidence of mcr-1 carriage in humans across China. Nat. Microbiol. 3, 1054–1062. doi: 10.1038/s41564-018-0205-8 30038311 PMC6198934

[B52] Song S. Wood T. K. (2020). A primary physiological role of toxin/antitoxin systems is phage inhibition. Front. Microbiol. 11. doi: 10.3389/fmicb.2020.01895 PMC7438911 32903830

[B53] Tacconelli E. Carrara E. Savoldi A. Harbarth S. Mendelson M. Monnet D. L.. (2018). Discovery, research, and development of new antibiotics: the WHO priority list of antibiotic-resistant bacteria and tuberculosis. Lancet Infect. Dis. 18, 318–327. doi: 10.1016/S1473-3099(17)30753-3 29276051

[B54] Trung N. V. Matamoros S. Carrique-Mas J. J. Nghia N. H. Nhung N. T. Chieu T. T. B.. (2017). Zoonotic transmission of mcr-1 colistin resistance gene from small-scale poultry farms, Vietnam. Emerging Infect. Dis. 23, 529–532. doi: 10.3201/eid2303.161553 PMC5382726 28221105

[B55] Wang J. T. Chang S. C. Chen Y. C. Luh K. T. (2000). Comparison of antimicrobial susceptibility of citrobacter freundii isolates in two different time periods. J. Microbiol. Immunol. Infect. 33, 258–262. 11269372

[B56] Wang C. Feng Y. Liu L. Wei L. Kang M. Zong Z. (2020). Identification of novel mobile colistin resistance gene mcr-10. Emerg. Microbes Infect. 9, 508–516. doi: 10.1080/22221751.2020.1732231 32116151 PMC7067168

[B57] Wang X. Wang Y. Zhou Y. Li J. Yin W. Wang S.. (2018). Emergence of a novel mobile colistin resistance gene, mcr-8, in NDM-producing klebsiella pneumoniae. Emerg. Microbes Infect. 7, 122. doi: 10.1038/s41426-018-0124-z 29970891 PMC6030107

[B58] Xavier B. B. Lammens C. Ruhal R. Kumar-Singh S. Butaye P. Goossens H.. (2016). Identification of a novel plasmid-mediated colistin-resistance gene, mcr-2, in escherichia coli, Belgium, June 2016. Euro. Surveill. 21, 27. doi: 10.2807/1560-7917.ES.2016.21.27.30280 27416987

[B59] Yang Y.-Q. Li Y.-X. Lei C.-W. Zhang A.-Y. Wang H.-N. (2018). Novel plasmid-mediated colistin resistance gene mcr-7.1 in klebsiella pneumoniae. J. Antimicrob. Chemother. 73, 1791–1795. doi: 10.1093/jac/dky111 29912417

[B60] Yin W. Li H. Shen Y. Liu Z. Wang S. Shen Z.. (2017). Novel plasmid-mediated colistin resistance gene mcr-3 in escherichia coli. MBio 8, e00543-17. doi: 10.1128/mBio.00543-17 28655818 PMC5487729

[B61] Zelendova M. Papagiannitsis C. C. Valcek A. Medvecky M. Bitar I. Hrabak J.. (2020). Characterization of the complete nucleotide sequences of mcr-1-Encoding plasmids from enterobacterales isolates in retailed raw meat products from the Czech republic. Front. Microbiol. 11. doi: 10.3389/fmicb.2020.604067 PMC7843963 33519748

[B62] Zhang R. Yang L. Cai J. C. Zhou H. W. Chen G.-X. (2008). High-level carbapenem resistance in a citrobacter freundii clinical isolate is due to a combination of KPC-2 production and decreased porin expression. J. Med. Microbiol. 57, 332–337. doi: 10.1099/jmm.0.47576-0 18287296

[B63] Zheng B. Xu H. Lv T. Guo L. Xiao Y. Huang C.. (2020). Stool samples of acute diarrhea inpatients as a reservoir of ST11 hypervirulent KPC-2-Producing klebsiella pneumoniae. mSystems 5, e00498-20. doi: 10.1128/mSystems.00498-20 32576652 PMC7311318

[B64] Zhou H.-W. Zhang T. Ma J.-H. Fang Y. Wang H.-Y. Huang Z.-X.. (2017). Occurrence of plasmid- and chromosome-carried mcr-1 in waterborne enterobacteriaceae in China. Antimicrob. Agents Chemother. 61, e00017-17. doi: 10.1128/AAC.00017-17 28559252 PMC5527621

